# Adaptive grip force is modulated by subthalamic beta activity in Parkinson's disease patients

**DOI:** 10.1016/j.nicl.2015.09.010

**Published:** 2015-09-29

**Authors:** Lukas L. Imbach, Heide Baumann-Vogel, Christian R. Baumann, Oguzkan Sürücü, Joachim Hermsdörfer, Johannes Sarnthein

**Affiliations:** a Department of Neurology, University Hospital Zurich, Frauenklinikstrasse 26, Zurich 8091, Switzerland; b Neuroscience Center, University of Zurich and ETH Zurich, Zurich, Switzerland; c Department of Neurosurgery, University Hospital Zurich, Frauenklinikstrasse 10, Zurich 8091, Switzerland; d Department of Sport and Health Sciences, Georg-Brauchle-Ring 60/62, Technische Universität München, München D-80992, Germany; e University of Zurich, Zurich, Switzerland

**Keywords:** Subthalamic nucleus, Motor control, EEG, Synchronization, Beta oscillations, PD, Parkinson's disease, DBS, deep brain stimulation, STN, subthalamic nucleus, ERD, event related desynchronization, ERP, event related potentials

## Abstract

**Introduction:**

Healthy subjects scale grip force to match the load defined by physical object properties such as weight, or dynamic properties such as inertia. Patients with Parkinson's disease (PD) show an elevated grip force in dynamic object handling, but temporal aspects of anticipatory grip force control are relatively preserved. In PD patients, beta frequency oscillatory activity in the basal ganglia is suppressed prior to externally paced movements. However, the role of the subthalamic nucleus (STN) in anticipatory grip force control is not known.

**Methods:**

After implantation of deep brain stimulation (DBS) electrodes in the STN, PD patients performed adaptive and voluntary grip force tasks, while we recorded subthalamic local field potentials (LFP) and scalp EEG.

**Results:**

During adaptive grip force control (Shake), we found event related desynchronization (ERD) in the beta frequency band, which was time-locked to the grip force. In contrast, during voluntary grip force control (Press) we recorded a biphasic ERD, corresponding to peak grip force and grip force release. Beta synchronization between STN and cortical EEG was reduced during adaptive grip force control.

**Conclusion:**

The time-locked suppression of beta oscillatory activity in the STN is in line with previous reports of beta ERD prior to voluntary movements. Our results show that the STN is involved in anticipatory grip force control in PD patients. The difference in the phasic beta ERD between the two tasks and the reduction of cortico-subthalamic synchronization suggests that qualitatively different neuronal network states are involved in different grip force control tasks.

## Highlights

• Subthalamic nucleus is involved in anticipatory grip force control in PD patients.
• Subthalamic beta activity is the first derivative of grip force in a shaking task.
• Synchronization between STN and cortex is reduced during the shaking task.

## 1 Introduction

Scaling and temporal adjustment of precision grip force is a highly efficient skill in everyday life. While grasping an object, healthy subjects precisely scale the applied grip force to match the load defined by physical object properties, such as weight and shape, as well as dynamic properties such as inertia (Prodoehl et al., 2009).

Neural implementation of precision grip force control is embedded in a complex network involving pre-motor cortical areas, the cerebellum and sub-cortical structures, particularly the basal ganglia (Nowak et al., 2007; Dafotakis et al., 2008; Prodoehl et al., 2009). Neuroimaging studies have shown that basal ganglia are involved in both predictive (dynamic) aspects of grip force control, as well as parameterization of grip force scaling (Vaillancourt et al., 2007; Prodoehl et al., 2008, 2009; Wasson et al., 2010).

In Parkinson's disease (PD) a distinction between dynamic grip force control and grip force scaling is observed: Whereas temporal aspects of dynamic grip force control are relatively preserved (Nowak and Hermsdörfer, 2002; Albert et al., 2010), grip force scaling is pathologically elevated in PD patients (Fellows et al., 1998). Direct evidence for the involvement of the subthalamic nucleus (STN) in grip force scaling has been obtained in PD patients treated by deep brain stimulation (DBS), where pathologically elevated peak grip force could be normalized by chronic DBS (Wenzelburger et al., 2002).

For temporal adaptation of precision grip force, the cerebellum is another key structure: It has been shown that patients with cerebellar disease suffer from impaired grip force control (Rost et al., 2005; Nowak et al., 2007). In this line, grip force adaptation relies on internal anticipatory models in the brain, which are mainly based in the cerebellum (Miall et al., 1993; Wolpert and Miall, 1996; Wolpert et al., 1998). The tight functional connections between basal ganglia and cerebellum (Hoshi et al., 2005; Bostan et al., 2010), suggest a dynamic interplay between the cerebellum and the basal ganglia in dynamic grip force control. While data from neuroimaging, anatomy and behavior point to an important role of basal ganglia networks in grip force control, the underlying neuronal activity is still unknown.

Various studies have demonstrated high beta power in the STN of PD patients (Brown et al., 2001) and the amount (Kühn et al., 2004; Pogosyan et al., 2010; Zaidel et al., 2010) and stability (Little et al., 2012) of beta activity in the STN correlates negatively with motor performance. The outstanding role of beta oscillations for bradykinesia has been demonstrated by inducing a frequency-specific impairment in a grip force task upon low-frequency stimulation in the STN of PD patients (Chen et al., 2011). Whereas beta activity in the basal ganglia may simply be an epiphenomenon of enhanced neuronal synchronicity during movement initiation, the suppression of beta activity before movement initiation in event-related tasks (Brown et al., 2001; Kühn et al., 2006; Oswal et al., 2012) provides evidence that dynamic changes in beta oscillations are critical for motor control *per se*. Extending this idea, dissociation of salient cues and actual motor execution supports the hypothesis that beta desynchronization prospectively modulates executive motor processing (Oswal et al., 2012; Gremel and Costa, 2013). To investigate prospective motor control, we examined how STN beta activity is modulated with adaptive grip force control during a shaking movement as compared to a control condition with voluntary grip-force initiation.

## 2 Methods

### 2.1 Patients and surgery

We included 6 PD patients who underwent DBS in the subthalamic nucleus (STN). Patients' demographic data and clinical details are summarized in Table 1. Bilateral DBS electrodes (Model 3389, Medtronic Neurological Division, Minneapolis, MN, USA) were implanted after MRI-based direct targeting of the STN (Bejjani et al., 2000). Intra-operatively, accurate implantation of the electrodes within the STN was verified by microelectrode recordings, followed by test stimulation to assess the clinical response, and by CT-imaging to reconstruct the effective electrode position (Schrader and Mehdorn, 2004). The data presented here were recorded on the second post-operative day at preoperative l-dopa levels (ON condition). Local field potentials (LFP) were recorded on temporarily externalized wires before implantation of the DBS impulse-generator. All patients gave informed written consent to participate in the study. The study was approved by the institutional ethics review board (*Kantonale Ethikkommission Zurich* KEK-ZH: 2012-0327).

### 2.2 Grip force recording

**Fig. 1:**
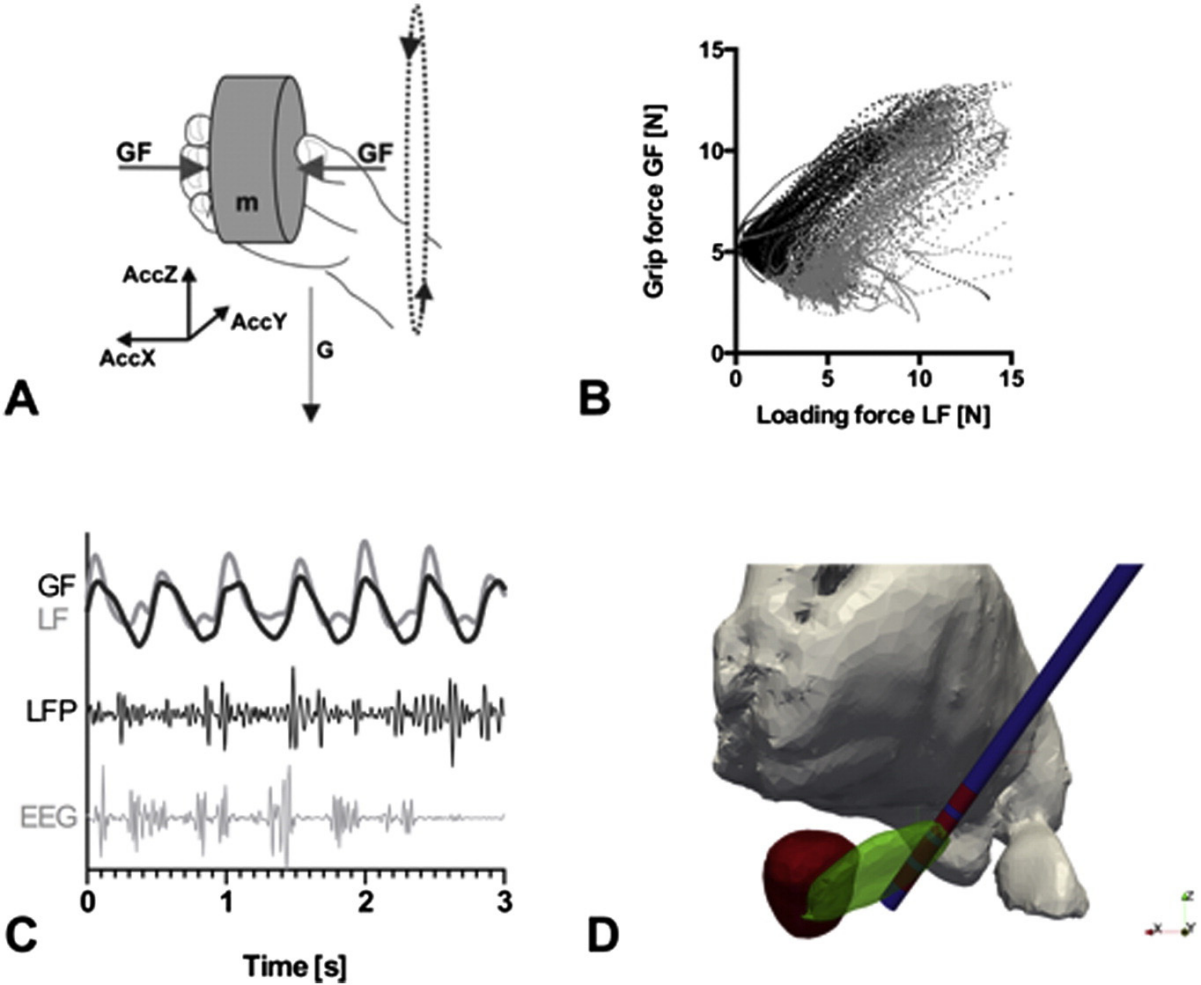
Experimental setup. (A) Schematic plot of the measuring device for simultaneous acquisition of grip force (GF) and acceleration in 3 dimensions (AccZ, AccX, AccY). Acceleration in the Z-direction (AccZ) equals the loading force (LF). (B) Illustration of the correlation between grip force and loading force (i.e. acceleration in Z direction) for upwards (black) and downwards (gray) directed movement in one patient during the shaking task (C) behavioral data (grip force GF and loading force LF) and electrophysiological data (local field potentials LFP and surface EEG) were recorded simultaneously. (D) Projection of the reconstructed electrode position onto a 3D-Atlas (Sarnthein et al., 2013). The second lowest contact (red) of the electrode (blue) is located in the dorsal STN (green) and this signal was used for subsequent analyses. Red nucleus (red) and thalamus (gray).

Adaptive grip force control during motor tasks was measured by a customized device. This device determines and records the applied grip force of the patient's fingers with an in-built force sensor and contains linear acceleration sensors for simultaneous registration of movement in three dimensions (Fig. 1A). In the case of oscillatory movements (e.g. shaking), force adaptation relies on an anticipatory internal model. Successful anticipatory grip force control is characterized by a matching of the applied grip force to the loading forces (mass + acceleration) of the device, which were generated by the movement. The device is cuboid (60 × 60 × 40 mm) and weighs 300 g (Fig. 1B) and emits a TTL pulse for synchronization with other data acquisition systems. To quantify the accuracy of the time-dependent grip-force adaptation, we calculated the correlation coefficient between grip force and loading force (Table 2) as a quantitative measure for the quality of grip force adaptation (Nowak and Hermsdörfer, 2005).

### 2.3 LFP and EEG recordings

The LFP was recorded from all contacts within both STN of each patient (sampling rate 200 Hz). Simultaneously, we recorded scalp EEG from a 12-channel subset of the 10–20 system at the fronto-polar (Fp1/Fp2), frontal (F3/F4), central (C3/C4), occipital (O1/O2) and midline (Fpz/Fz/Cz/Oz) electrode sites (Fig. 1C). The central midline electrode Cz was used as recording reference for EEG and LFP. As verified by post-operative reconstruction of the electrode position, the second lowest contact (Fig. 1D, Sarnthein et al., 2013) was located in the motor part of the STN in all patients and taken for further analysis. To reduce movement and electrode artifacts, we digitally re-referenced all signals to a Laplacian montage with weighted averages of the surrounding deep brain electrodes (for LFP channels) and surface electrodes (for EEG channels). This montage allowed for a significant reduction of the artifact level, but at the same time ensured the linear independence of cortical and LFP signals for the calculation of cortico-subthalamic synchronization.

### 2.4 Motor tasks

All experiments were performed in a sitting position. Patients grasped the measurement device with all fingers of one hand, while the other arm was in a resting position. To minimize interference with visual feedback, all experiments were performed with closed eyes.

For the shaking task (Shake), patients were instructed to shake the cube in a predefined manner, i.e. to perform consecutive point-to-point up- and downward movements in front of the trunk with an amplitude of about 20 cm. This shaking movement was self-paced, but patients were instructed to reach a frequency of approximately 2 Hz, if possible, depending on bradykinesia and rigor. After instruction of the patients and test-runs where necessary, we recorded a 90 s-epoch for each hand.

Two control tasks were performed: In a hold condition (Hold), the device was held steadily in one hand without movement to measure the ‘resting state’ background level of the STN and cortical EEG signal. For a pressing condition (Press), patients pressed rhythmically on the device in the same frequency as the arm was moved during the shaking task, but without moving the device itself. This task was introduced to control for *voluntary* self-paced grip-force initiation (Press), as compared to *anticipatory* grip force control adjusted by somatosensory feedback (Shake). By this experimental design we were able to compare two movements with identical grip force (i.e. rhythmic contraction of the fingers in one hand), but presumably different central activating network states.

**Fig. 2:**
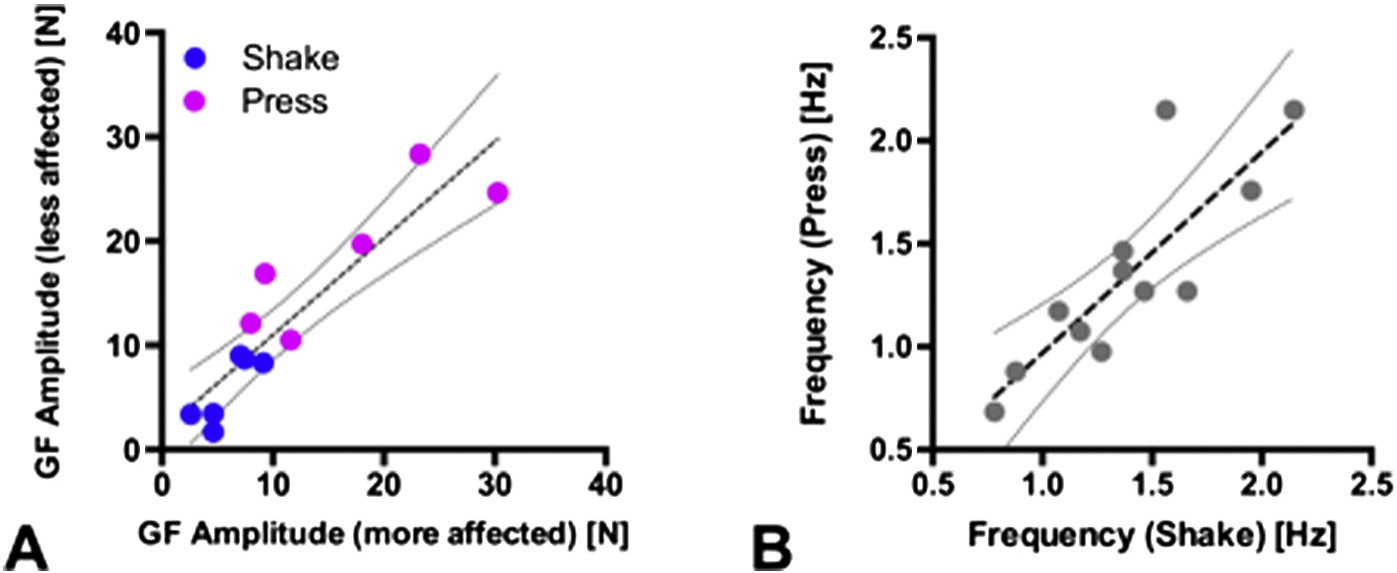
Grip force amplitude and frequency during motor tasks. (A) Analysis of the mean GF amplitude for the more affected, versus the less effected hand in all patients showed a significant correlation, indicating that GF amplitudes did not differ between both extremities within subjects (Slope: 0.92 ± 0.13, R^2^ = 0.82, p < 0.005, N = 12). Across tasks, the mean grip force was higher during Press (magenta, 17.7 ± 7.2 N) than during Shake (blue, 5.8 ± 2.7 N, p < 0.005 paired t-test). (B) The mean movement frequency of Shake and Press was highly correlated across hands (Slope: 0.97 ± 0.19, R^2^ = 0.71, p < 0.005, N = 12, dotted lines: 95% confidence intervals for linear correlation).

All subjects performed the tasks in the same sequence Hold–Shake–Press. Hold familiarized the subjects with the cube. In Shake, subjects discovered and trained their individual shaking frequency. Subjects then used this frequency in the self-paced Press condition (Fig. 2B).

To compare the motor output of the clinically more affected with the less affected side, we calculated the mean amplitude of the applied grip force for both hands during the motor tasks. To test for a disease specific impairment, we correlated the mean grip force amplitudes from both sides in all patients (N = 12 recordings, Fig. 2A). The mean frequencies of both movements (Press and Shake) were determined by spectral analysis of the grip force trace (Welch's periodogram; 2000 ms non-overlapping Hanning window). The peak frequency for Press was then correlated with the peak frequency of Shake (Fig. 2B).

The load force was calculated as the sum of weight (m × G), acting vertically to the grip surface, and the acceleration-dependent inertial loads in the vertical and sagittal directions (m × AccZ, m × AccY) (Rost et al., 2005).

### 2.5 Spectral power

We computed the power spectral density (PSD) of the LFP with Welch's periodogram on the full 90 s epoch for all conditions (1000 ms non-overlapping Hanning window). For all consecutive analyses of the LFP signals, we correlated the LFP recording with the behavioral data from the contralateral hand in all patients.

The event-related analysis was conducted following the approach by Kühn and coworkers (Kühn, 2004). For Shake and Press, the maximal grip force of each cycle was used as a trigger event. Event-related potentials (ERP) were calculated for 250 ms before and after this event: the LFP signal was beta-bandpass-filtered (13–35 Hz), amplitude-squared and averaged across all events within one subject. To get a comparable measure of time-dependent beta activity, the normalized cumulative sum (Kühn, 2004) of the beta ERP was calculated. This measure provides ascending slopes during phases of beta-synchronization (high average beta activity), and descending slopes for beta-desynchronizing states (low average beta activity). The resulting time-dependent signals were averaged across subjects and hemispheres. Confidence limits are given as a standard error of the mean (SEM) for all averaged spectra and ERP (N = 12).

### 2.6 Synchronization measures

The synchronization between EEG and LFP was first estimated by magnitude squared coherence (MSC) between the LFP and the ipsilateral central EEG-derivation (1000 ms non-overlapping Hamming window). The magnitude squared coherence MSC_*xy*_ for two signals, *x* and *y*, is equal to the average cross-power spectrum P_*xy*_ normalized by the averaged power spectra of the signals MSC_*xy*_ = |P_*xy*_|^2^ / (P_*xx*_P_*yy*_). Coherence assesses the strength of the linear relationship between two signals at every frequency f, and its value lies between 0 and 100%. It estimates the degree to which phases and amplitudes are dispersed at the frequency of interest. MSC_*xy*_ = 0 means phases and amplitudes are randomly dispersed among all epochs. Signals are perfectly coherent (MSC_*xy*_ = 100%) at a given frequency when they have both a constant phase difference φ and constant amplitude ratio over the time considered. In this case, phases of signals *x* and *y* are identical in all epochs (i.e. the two signals are completely phase-locked at this frequency). The time lag between EEG and LFP was estimated on the basis of the phase differences φ of the cross-spectral power as calculated by Welch's averaged periodogram (1000 ms non-overlapping Hanning window).

To compare EEG–LFP synchronization across different motor behaviors, we then calculated the phase locking value (PLV) (Tass et al., 1998). We chose the PLV because – as opposed to MSC – it is independent of signal amplitude and is therefore more reliable for the direct comparison of different motor tasks, which elicit markedly different power spectral densities in the beta band. Taking the frequency range where the MSC differed most across motor tasks, we subsequently calculated the PLV in the high beta band (20–35 Hz).

### 2.7 Statistics

Motor output was analyzed with code written in LabView (Nowak and Hermsdörfer, 2005). Spectral analyses were performed with custom scripts written in MatLab (http://www.mathworks.com). For the calculation of the PLV we used the Neurophysiological Biomarker Toolbox (Hardstone et al., 2012). We used GraphPad Prism (http://www.graphpad.com) for statistical analyses and to create the figures. Parametric and non-parametric tests were used as applicable. Statistical significance was established at p < 0.05.

## 3 Results

### 3.1 Motor output

To investigate the time-dependent adaptation of grip force during the shaking task, we analyzed the correlation between loading force and applied grip force as illustrated in Fig. 1B. The correlation coefficients in the first (grip force increase) and second (grip force release) phase of the movement did not differ significantly (Table 2). The motor output of grip force adaptation was thus the same for both tasks.

Grip force amplitude of the clinically more affected and the clinically less affected hand were highly correlated (Slope: 0.92 ± 0.13, R^2^ = 0.82, p < 0.005, Fig. 2A), indicating that the patients were able to perform the grip force tasks with each hand at the same precision. Across tasks, the mean amplitude was significantly higher during Press than in Shake (p < 0.005, Fig. 2A).

The mean movement frequency during Shake and Press showed a highly significant correlation, indicating that the movement frequencies were stable within one hand (Slope: 0.97 ± 0.19, R^2^ = 0.71, p < 0.005, Fig. 2B).

### 3.2 Beta power in STN

**Fig. 3:**
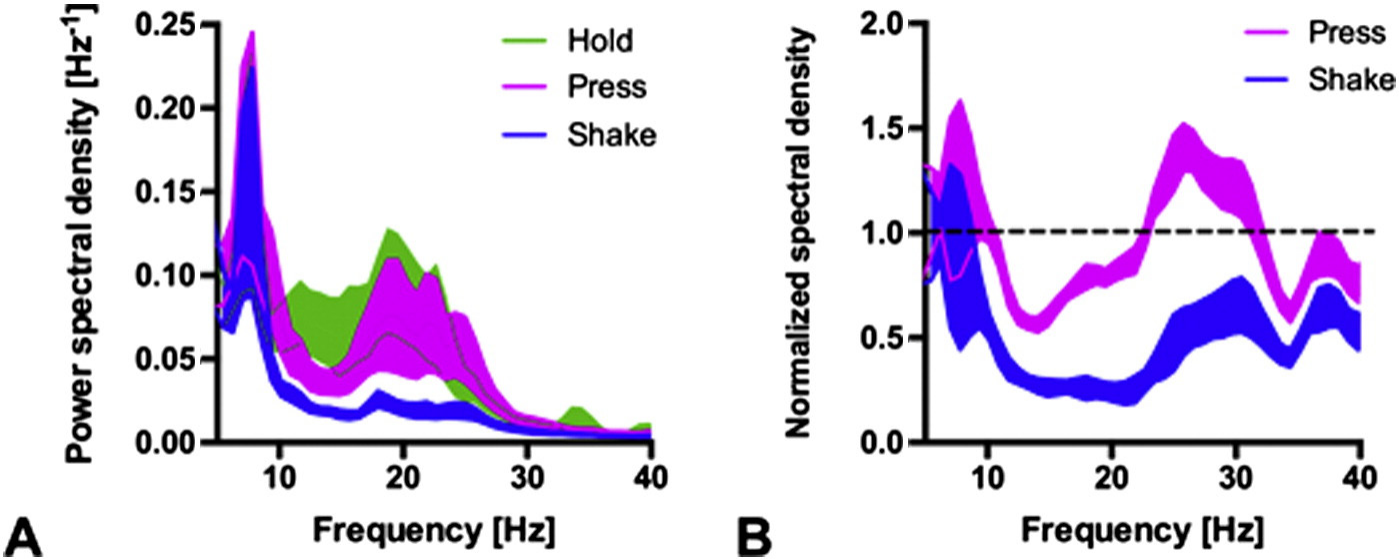
Task-specific beta oscillatory activity. (A) The power spectral density (PSD) averaged across hands (N = 12) showed a broad beta peak during Hold (ribbon: SEM). (B) Normalizing the PSD of Shake and Press by the PSD of Hold reveals some beta decrease for Press and a pronounced beta desynchronization during Shake.

During the Hold condition, the mean PSD of the STN traces showed a high beta band activity with a peak frequency around 20 Hz (Fig. 3A). To illustrate the PSD-reduction across hands (N = 12), we normalized the PSD of Shake and Press by the PSD of Hold (Fig. 3B). This revealed some changes in beta during Press and a pronounced beta desynchronization during Shake.

### 3.3 Event related beta desynchronization in STN

**Fig. 4:**
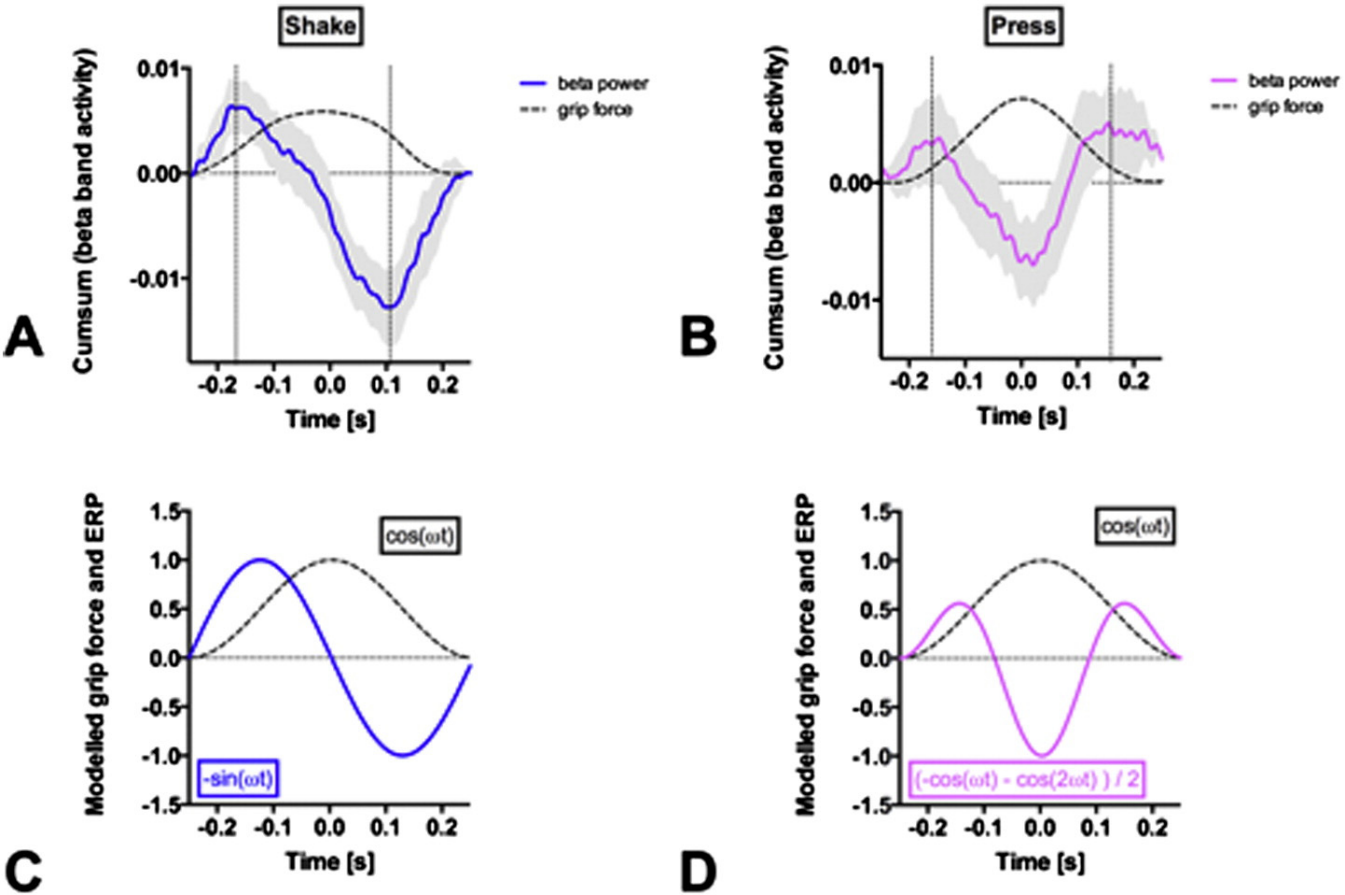
Grip force and beta ERP in STN. (A) Shaking task traces. The averaged grip force (dashed line) increases towards t = 0 while subjects move the device downwards. For averaging, grip force curves were aligned at maximal grip force at t = 0 (reversal point). The averaged grip force curve turns to the right (second derivative <0) between infliction points (vertical dashed lines) and beta ERP (blue line; gray ribbon: SEM for N = 12) decreases monotonically. (B) Pressing task traces. While the grip force evolves as that during Shake, the beta ERP (magenta line; gray ribbon: SEM for N = 12) follows a biphasic pattern. (C) Modeling Shake traces. The grip force trace follows cos ωt with frequency ω. The time course of STN beta ERP is the derivative of the applied grip force, d(cos ωt) / dt = −sin ωt. (D) Modeling Press traces. The grip force trace follows cos ωt as that during Shake. The time course of STN beta ERP (−cos ωt − cos 2ωt) / 2 is proportional to a superposition of the second order derivatives d(cos ωt)^2^ / dt^2^ at the fundamental frequency (ω) and the first harmonic (2ω).

To investigate the time course of beta event related potentials (ERP), we defined the maximal grip force of each movement cycle as the trigger event. During Shake, the beta power in STN evolved in a sine wave, which was indeed tightly time-locked to the phase of the grip force (Fig. 4A). The maximal beta desynchronization occurred at the time point when the grip force curve had its maximal slope, i.e. at the inflection point preceding maximal grip force (downward movement). The time course of STN beta ERP during Shake can thus be modeled as the derivative of the applied grip force, d(cos ωt) / dt = −sin ωt (Fig. 4C). In other words: The incremental change of force is proportional to the change in beta ERP.

During Press, STN beta ERP followed a two-phasic pattern. The two peaks of beta ERP occurred near the two inflection points of the grip force curve, one preceding maximal grip force and one preceding minimal grip force (Fig. 4B). The first peak of Press has the same amplitude and time-lag (−0.15 s) as the peak in Shake. In Fig. 4D, modeling the time course of STN beta power by (−cos ωt − cos 2ωt) / 2 gives 0 at t = −π and −1 at t = 0 zero with the periodicity and the symmetry of the data in Fig. 4B. The model function is proportional to a superposition of the second order derivatives d(cos ωt)^2^ / dt^2^ at the fundamental frequency (ω) and the first harmonic (2ω).

### 3.4 Synchronization between LFP and scalp EEG

**Fig. 5:**
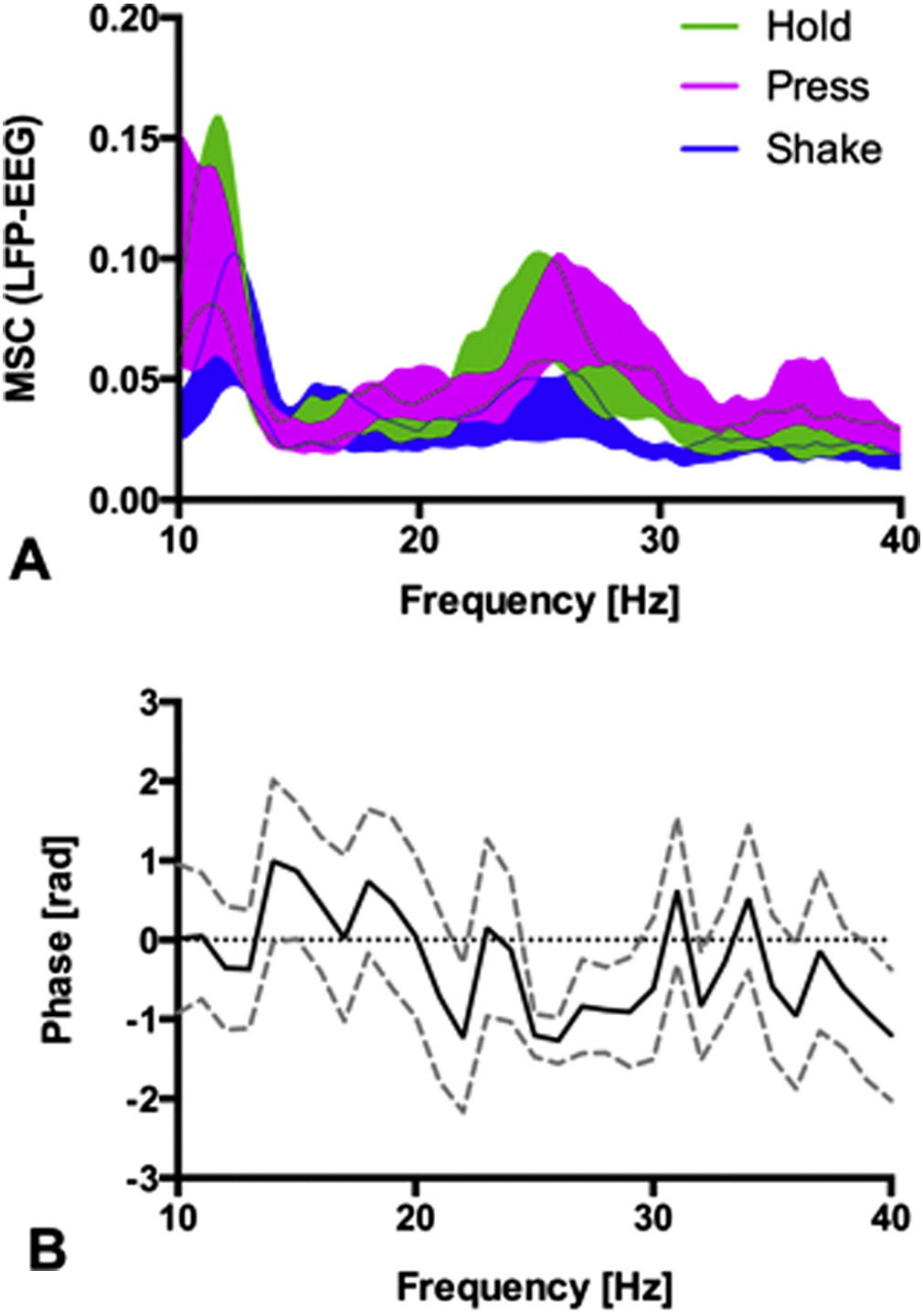
Coherence and phase lag between LFP and EEG. (A) Magnitude-squared coherence (MSC) during Shake (mean ± SEM, N = 12) is lower than that during Hold and Press in high beta (20–35 Hz). (B) The phase spectrum (mean ± SEM, N = 12) is negative in the high beta range (phase lag = −1.3 rad). Scalp EEG leads the LFP.

Fig. 5A shows the MSC between LFP in the STN and the ipsilateral central EEG (C3 or C4, respectively) averaged across all subjects for all task conditions. The MSC with its broad beta peak (20–35 Hz) for Hold and Press resembles the PSD (Fig. 3A). As in the PSD, this MSC peak was reduced during Shake. Complementary to MSC, the time lag between LFP and EEG can be estimated from the phase spectrum. The time lags for individual patients are given in Table 3 (median 15 ms, SEM 5 ms). The averaged phase spectrum is given in Fig. 5B with mean beta phase lag −1.3 rad. Scalp EEG leads the subthalamic LFP.

**Fig. 6:**
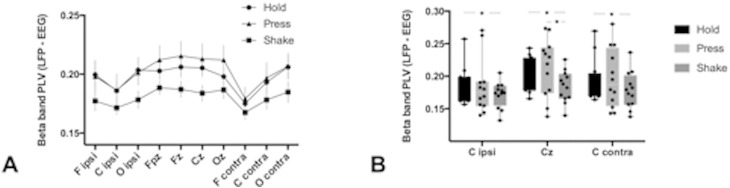
Phase locking (PLV) between LFP and EEG in high beta (20–35 Hz). (A) The PLV during Shake is lower than that during Hold and Press for all scalp electrode sites (ipsi: ipsilateral to STN; contra: contralateral to STN). (B) For central scalp electrodes, the PLV is lowest during Shake in all N = 12 recordings (* = p < 0.05, paired Wilcoxon test).

To compare the synchronization between STN and cortex across different tasks, we calculated the PLV between LFP and all EEG electrode sites (Fig. 6A). Guided by the frequency range of high MSC, we calculated the PLV in the 20–35 Hz high beta band. The PLV was high during Hold and Press and was reduced during Shake. The reduction of PLV was most pronounced over the midline sites (Fig. 6B).

## 4 Discussion

### 4.1 Adaptive grip force control involves STN activity

We investigated the role of beta oscillatory activity in the STN during adaptive grip force tasks in PD patients. The core finding was that the LFP in the STN during temporal grip force adaptation showed a time-locked beta band desynchronization in all subjects and hands. The LFP spectra showed marked differences between two movement tasks (Shake vs. Press), although the behavioral motor output (the temporal adaptation of precision grip force) was the same in both tasks (Fig. 4). In other words: for the same motor behavior (rhythmic compression and release of the device upon shaking or pressing), two distinct electrophysiological patterns were observed in the contralateral STN. This finding indicates that the basal ganglia are involved in voluntary and adaptive precision grip force control, and that the type of motor behavior crucially affects the network processing of grip force adaptation in the basal ganglia.

In agreement with previous studies in PD patients, ongoing movement desynchronized STN beta power compared to the resting state (Hold). The desynchronization was more pronounced for the habitual grip force task (Shake, Fig. 4A) as compared to the voluntary grip force task (Press, Fig. 4B). Similarly, cortico-subthalamic synchronization (MSC and PLV) was markedly reduced during Shake (Figs. 5 and 6). This suggests that a habitual, internalized and over-learned motor task like shaking the device is controlled with less cortical involvement.

### 4.2 PD patients are not impaired in the Shake task

For the Shake task, PD patients' behavior is comparable to that of healthy controls as has been demonstrated previously by the high correlation of adaptive grip force to a temporal changing loading force (Nowak and Hermsdörfer, 2002; Albert et al., 2010). Also in our patient group, motor output and electrophysiological characteristics were the same for the more affected and the less affected brain hemisphere. This suggests that the observed dynamic STN modulation might be representative of any STN including a healthy one.

### 4.3 Adaptive grip force control relies on an internal model

From a computational perspective, we can interpret these findings in the light of the proposed forward models during cerebellar-driven precision grip force tasks. Currently, in this field there is still very little behavioral and electrophysiological data in humans regarding the proposed interplay between the basal ganglia and the cerebellum. Only two studies provide evidence that the anatomical connections between the cerebellum and the basal ganglia are also functionally relevant (Hoshi et al., 2005; Bostan et al., 2010). Based on the theoretical framework of internal forward models for cerebellar-generated movements (Wolpert and Miall, 1996; Wolpert et al., 1998), we propose that the two examined tasks in this study (Shake and Press) are processed by different neuro-anatomical networks.

During the Shake movement sequence, the downward movement is triggered voluntarily. There is only one beta ERD per movement cycle, suggesting that only the downward movement is initiated voluntarily. The upward movement, as a rebound, proceeds without STN beta ERD in this habitual movement sequence. The Shake movement sequence is highly habitual with little cortical involvement, as evidenced by the low cortico-subthalamic synchronization (Figs. 5 and 6). Adapting the grip force does not consume conscious resources but is rather derived from an internal model, which involves the cerebellum. In the STN, the instantiation of the internal model is reflected in the mathematical first derivative of the grip force d(cos ωt) / dt (Fig. 4C). The subthalamic beta ERP thus represents not the force itself but rather the incremental change of force. This supports the role of the basal ganglia as a dynamic relay in fine-tuning of motor execution.

In the Press task, both the grip force initiation and the grip force release are triggered voluntarily. We found cortico-subthalamic synchronization as high as during resting (Hold, Figs. 5 and 6) and two peaks of STN beta ERP in the movement cycle. The two peaks are reflected in the mathematical harmonic (2ω) and the second order derivative d(cos ωt)^2^/dt^2^ in our model of the beta time course (Fig. 4D).

### 4.4 Anatomical considerations

On a network level, the reduced coherence during the shaking task can be interpreted in light of the proposed neuroanatomical distinction between grip force scaling and temporal grip force control in anterior and posterior basal ganglia nuclei: for the voluntary Press task we found higher cortico-STN coherence, indicating that this task is embedded in a cortico-basal ganglia network controlling for grip force *parameterization*. On the other hand, *adaptive* grip force control is mediated predominantly through anterior basal ganglia nuclei and accordingly cortico-STN coherence is diminished during Shake. High cortico-STN connectivity during Press may also point to an involvement of the hyperdirect pathway in voluntary grip force control. In this light, our findings suggest that the hyperdirect pathway is predominantly activated during voluntary grip force control and reduced in adaptive grip force control. Furthermore, the hyperdirect pathway has been proposed to play a role in sustaining beta oscillatory activity in the STN (Jenkinson and Brown, 2011; Moran et al., 2011). Accordingly, we found higher power spectral density in the beta band during the pressing task, supporting the argument that an (over-)active hyperdirect pathway may cause elevated beta band activity in the STN. Finally, on a behavioral level, we observed significantly higher grip force amplitudes during the pressing task as compared to the shaking task, which could be caused by inhibiting signals from the hyperdirect pathway during voluntary grip force control.

### 4.5 Implications for the understanding of dysfunctions in PD patients

STN beta ERP showed a different time course in the two movement tasks. In Shake, there was one peak of beta ERP prior to maximal grip force. In Press there were two peaks, one prior to maximal and one prior to minimal grip force. These temporal changes of synchronicity in the basal ganglia provide complementary information in the understanding of pathological network activity in PD patients (Little et al., 2012).

As a clinical observation, habitual movement control is typically more affected in PD patients. Assuming that the baseline beta oscillatory activity (like Hold) is pathologically elevated in PD patients, a higher amount of beta desynchronization is needed to initiate and maintain habitual movement (like Shake), which is prominently controlled by sub-cortical networks. Clinical studies showed a prolonged and excessive grip force adaptation after motor engagement (Wenzelburger et al., 2002). This is reflected by the observation that not only movement initiation is impaired in PD patients (as seen, e.g. in freezing of gait), but also termination of an ongoing movement is disturbed, resulting in involuntary prolonged movements (e.g. festinating gait). Similarly to this behavioral evidence of reduced control in motor dis-engagement in PD patients, the electrophysiological investigation of beta oscillations also show a marked difference exactly in the release phase of the cyclic movement, where the second beta ERD is not seen during the shaking task (Fig. 3). In this light, our findings could also be interpreted as an electrophysiological correlate of impaired movement termination: During Shake, no beta ERD was measured when grip and load force decreased during the upward movement. Therefore, the missing beta desynchronization in the late phase of the cycling movement could be interpreted as a correlate for the reduced ability for movement termination in PD patients.

Similarly, the interplay between motor cortex and basal ganglia (as measured by PLV) was significantly reduced during Shake as compared to Press. When the functional connectivity to the motor cortex is high (as in Press) the temporal cueing in the basal ganglia seems to be more precise and more adaptive, as compared to Shake, where cortico-STN correlation is lower and therefore the temporal change in beta desynchronization during an ongoing movement is less adaptable.

## 5 Conclusions

The time-locked suppression of beta oscillatory activity in the STN is in line with previous reports of beta ERD prior to voluntary movements. Our results show that the STN is involved in anticipatory grip force control in PD patients. The difference in the phasic beta ERD between the two tasks and the reduction of cortico-subthalamic synchronization suggests that qualitatively different neuronal network states are involved in different grip force control tasks.

## Conflicts of interest

The authors declare that there are no conflicts of interest.

## Funding

This study was investigator-sponsored (JS).

## Figures and Tables

**Table 1:** Demographic and clinical patient characteristics. UPDRS: Unified Parkinson's Disease Rating Scale, ON/OFF values of preoperative l-dopa challenge test; LED: levodopa equivalent dose at the time of recording.

ID	Age [y]	Gender	Parkinson type	Disease duration [y]	Hoehn–Yahr Scale	UPDRS III ON/OFF	LED [mg/d]
1	61	F	Rigid akinetic	9	2	12/24	850
2	63	M	Tremor dominant	12	2	16/55	1000
3	48	M	Young onset	10	2.5	11/53	500
4	47	M	Young onset	12	2	18/46	1000
5	73	M	Tremor dominant	10	2	18/37	1000
6	53	M	Rigid akinetic	14	2.5	28/52	2300

**Table 2:** Correlation coefficients (Pearson's R) for grip force and loading force in fixed intervals of 250 ms during grip force release (upward movement) and grip force increase (downward movement).

Subject ID	GF increase	GF release
1	0.44	0.43
2	0.74	0.58
3	0.93	0.94
4	0.49	0.58
5	0.73	0.68
6	0.66	0.84
Median	0.70	0.63

**Table 3:** Beta peak frequency, magnitude squared coherence (MSC), peak phase lag, and estimated time lag. In all subjects (N = 6), values of both hemispheres were averaged.

ID	Frequency [Hz]	MSC	Phase [rad]	Time lag [ms]
1	16	0.07	−0.6	−6
2	22	0.07	−2.8	−21
3	27	0.12	−1.2	−10
4	20	0.02	−1.9	−15
5	21	0.01	−1.9	−15
6	25	0.17	−3.1	−20
Median	**21.5**	**0.1**	**−1.9**	**−15**
